# Health aspects of Arctic exploration – Alaska’s medical history based on the research files of Dr. Robert Fortuine

**DOI:** 10.3402/ijch.v72i0.21264

**Published:** 2013-08-05

**Authors:** Kathleen Murray

**Affiliations:** Alaska Medical Library, University of Alaska Anchorage, AK, USA

**Keywords:** medical history, arctic bibliography, health care delivery, access to health care, Alaska

## Abstract

**Background:**

Robert Fortuine provided basic medical care to Alaska Native people, chronicled the Health Aspects of Arctic Exploration and through a number of influential publications, was the first to thoroughly document and analyse Alaska’s Medical History. This overview of his published work will provide the reader with a detailed overview, so that they can begin to explore Dr. Fortuine’s many published works in more detail.

**Objective:**

This review will explore Alaska’s Medical History and the Health Aspects of Arctic Exploration through the research files and the 10 most significant publications of Dr. Robert Fortuine.

**Design:**

Review of Dr. Fortuine’s major works and the master bibliography has over 3,000 references and 81 subjects. The master bibliography is a merger of 55 separate bibliographies, which provides a wealth of bibliographic information. This paper will describe his 10 most significant publications, 2 of which began as a journal issue.

**Results:**

Dr. Fortuine was a prolific writer throughout his career, publishing 134 articles and books. He wrote papers and books on Alaska’s medical history, tuberculosis and health care delivery from Russian–America through the Public Health Service efforts in the territory and then the State of Alaska. The master bibliography has over 3,000 references and 81 subjects. This list has a significant number of entries for tuberculosis with almost one-third of the entries including this heading. Others dwell on the history of “pre-contact” health, the history of Alaska Native health care, the history of the Alaska Department of Health, especially the tuberculosis programme, the role of the US Public Health Service and traditional medicine. He completely reviewed every Governors’ and the US Surgeon General’s reports in regard to Alaska content. This paper describes his 10 most significant publications.

**Conclusions:**

Robert Fortuine’s published works offer a wealth of information and insight into Alaska’s Medical History and the Health Aspects of Arctic Exploration. As is probably true for many historians, he began small, creating a bibliography and adapting a talk before tackling his first full-length book. Readers who sample his many works will be enriched and enlightened.

Alaska is a young state – turning 50 years old in 2009. Its medical history covers a much longer period of time, and this has been the lifelong interest of a physician and medical historian, Robert Fortuine. After introducing this amazing man, the remainder of this paper will touch upon his most substantial works.

## Background

Dr. Fortuine attended medical school in Montreal, Canada. He then served as a Commissioned Officer of the United States Public Health Service (USPHS) for 26 years. While on active duty, he earned a Master of Public Health degree from Harvard and completed residency in general preventative medicine at the University of Oklahoma’s Health Center. His duties took him to Arizona and then Alaska where he was a Service Unit Director of the Kanakanak (1963–1964) and Bethel hospitals (1964–1967), before becoming Director (CEO) of the Alaska Native Medical Center, Anchorage for 6 years (1971–1977). He was then detailed to the US State Department as international health attaché, to the World Health Organization from 1977–1980. He retired with the rank of captain in 1987.

He began his second career as a clinical teacher with the WWAMI program, which is a collaborative medical school among universities in 5 north-western states, Washington, Wyoming, Alaska, Montana and Idaho and the University of Washington’s School of Medicine. Dr. Fortuine instructed first year medical students in the WWAMI program. He officially retired in 2004; however, he continued to teach for another 5 years! There is a scholarship in his name to support Alaskan residents, particularly Alaska Natives, entering this programme.

He was also a Fellow of the Arctic Institute of North America, a founding member of the American Society for Circumpolar Health and a co-founder of the Amundsen Educational Center in Soldotna (a Christian vocational school for Alaska Natives). Over the course of his career, he received several Public Health Service medals and an honorary degree:

- The John Phillips Award “for service to the public good” given annually by the Phillips Exeter Academy to an alumnus whose life demonstrates the “ideal of goodness and knowledge united in noble character and usefulness to mankind”.
- The Trudeau Award given annually by the American Lung Association to an individual with lifelong major contributions to prevention, diagnosis and treatment of lung disease through leadership in research, education or clinical care.
- The Jack Hildes Medal awarded by the International Union for Circumpolar Health to an individual demonstrating excellence in northern medicine and health, in consideration of their contributions through service, research and humanitarianism.
- An Honorary Doctor of Science from the University of Alaska.

## Design

Dr. Fortuine was a prolific writer throughout his career, publishing 134 articles and books. He wrote papers and books on Alaska’s medical history, tuberculosis and health care delivery from Russian–America through the Public Health Service efforts in the territory and then the State of Alaska. This paper will describe his 10 most significant publications, 2 of which began as a journal issue.

## Discussion

As is probably true for many historians, he began small, creating a bibliography and adapting a talk before tackling his first full-length book.

His first significant publication is a bibliography, *The health of the Eskimos; a bibliography, 1857–1967* (1), which became the basis for a later bibliographic publication on the same topic. In this short work, he organises the entries beginning with historical and folk medicine, then studies on healthy individuals, followed by health programmes and surveys. The largest section covers infectious and non-infectious diseases. He notes in the introduction the extremely low incidence of diabetes and heart disease. Later, he collaborated with individuals and units of the University of Alaska Anchorage to produce *The health of the Inuit of North America: a bibliography from the earliest times through 1990* (2). He expanded on his first work and has created a schema for describing health issues of the Inuit of Canada and Alaska. This bibliography contains 2,742 items including books, book chapters, journal articles, proceedings, abstracts, personal accounts, research reports and a limited number of unpublished writings. Every item in this book is also in the Arctic Health Publications database found on the web at http://www.arctichealth.org.

Dr. Fortuine’s next publication was an adaptation of a talk given to the Oklahoma Public Health Association on April 18, 1975. It was published in the final issue of *Polar Notes* as the *Health care and the Alaska Native: some historical perspectives* (3). In this work, he discusses the health of natives before European contact:

Many persons cherish the belief that before the Bering and Chirikoff expeditions touched on Alaska’s shores in 1741, the native people lived in a pristine state of good health. The evidence shows this to be a serious oversimplification, although health conditions undoubtedly did worsen after the first European contacts. [p. 3]

He goes on to explain that endemic diseases, such as parasitic diseases, have always been present. Environmental disorders such as drowning, cold exposure and food safety (botulism or paralytic shellfish poisoning) continue today. However, introduced diseases have shaped the history of this people and the State. These diseases began with syphilis, followed by influenza, but the most devastating has been tuberculosis, which reached its zenith shortly after World War II. The focus of his paper was on how the Alaska Native health care system was developed with a major role played by the federal government. He concludes with noting that the “Alaska Native Health Service is a complex but workable system of comprehensive health which is unique in the United States and has even been considered a model for sparsely settled areas elsewhere in the world.”

Dr. Fortuine’s wide ranging interest in Alaskan history, and his fluent German, led him to translate *The Alaska Diary of Adelbert von Chamisso: Naturalist on the Kotzebue Voyage, 1815–1818* (4) from German. The diary is a personal account of the voyage written with an informal point of view and includes only the portion of Chamisso’s work describing his time in Alaska. Chamisso secured a position as a scientist on the *Rurik*, a small ship with a crew of 20 sailors. This small ship circumnavigated the world spending part of the summer of 1816 in Alaska before heading south, for what is now Hawaii, in September. They returned the following year reaching Alaska in mid-April and exploring until the middle of August. The crew of 20 sailors was led by Lt. Otto von Kotzebue and included 2 officers, Lts. Shishmarev and Zacharin-Kotzebue and Shishmarev are familiar names to Alaskans. The remaining crew was comprised of 2 petty officers, 3 second officers, a physician, an artist and 2 naturalists.

Given Dr. Fortuine’s interest in the history of how health care was delivered in Alaska, it is not surprising that he was asked to write a history about one of the major hospitals in the state. As director (CEO) of the Alaska Native Medical Center from 1971–1977, he was able to use interviews, personal records and official hospital files. *Alaska Native Medical Center: A History 1953–1983* (5) shares the evolution of a hospital built in 1953 as a tuberculosis (TB) hospital. In 1954, the TB incidence among Alaska Natives was 2,452.4 per 100,000. The US incidence in the same year was 62.4 per 100,000. This difference is a ratio of nearly 40:1. Within 10 years, the patient population had changed. One-third of the beds were still occupied by TB patients, while another third were reserved for paediatric patients. He continues to show the growth and development of different services through 4 decades. This work will interest anyone researching the structure, function and management of hospitals of this era.

After this detailed history about the impact of modern medicine on the healthcare of the Native population, Dr. Fortuine went back to a topic he returned to again and again – the value and use of traditional healing methods amongst the Alaska Natives. *The Use of Medicinal Plants by the Alaska Natives* (6) describes the use of traditional medicine by each of the Alaska Native groups: Aleut, Koniag, Chugach, Yupik and Inupiat Eskimos, Tlingit, Haida, Tsimshian and Athapaskans. It was published as an issue of the Alaska Medicine journal. He begins by acknowledging how potentially misleading the findings might be but concludes that there is still valuable information in this document. He finishes this paper with 5 recommendations:

- Perform chemical analyses of more plants.
- Document traditional use of healing plants: vital information is being lost as memories fade and traditional healers die.
- Encourage traditional healers to permit extensive interviews on their knowledge and use of plants for healing.
- Consider traditional healers as colleagues and integrate them into the health system – encourage training in clinical assessment similar to training provided to Community Health Aides.
- Encourage physicians, public health nurses and other practitioners to be more sensitive to the role that traditional healers play in Alaska Native society.

He was named Alaska Historian of the Year in 1990 for *Chills and Fever: Health and Disease in the Early History of Alaska* (7). He organises this work into sections: part 1 relates the health of the Alaska Natives at the time of contact; part 2 covers health issues in the early history of Alaska from the early explorations of Russians to the Nome gold rush; and part 3 discusses significant diseases and epidemics. In part 2, Dr. Fortuine recounts the health care system put in place by the Russian–American Company, which lasted almost 70 years and provided healthcare throughout the territory. This health system ended when Alaska was sold to the United States. Provision of health care services worsened with extremely limited access provided by the US Army, then Navy and then the Treasury Service – from their Revenue cutter ships. The role of educators in providing healthcare is recounted. One example is that of Dr. Sheldon Jackson who raised private funds in 1891 and purchased 16 reindeer, importing them from Siberia as a source of food and to become a new industry for the Alaska Native population. All of the reindeer died and 5 more trips to Siberia followed with 171 additional animals imported. Medically, this is of interest because 2 staff members were physicians. The reindeer station physicians provided care not only to the imported Lapp herders, but also to Native patients, miners and sick reindeer! This part concludes with the earliest missionary efforts of Russian Orthodox and Roman Catholic men and women, followed by Presbyterian, Episcopal and other protestant groups. While much of their work was positive, a major war was waged against the role of shamanism as a form of medical treatment.

Dr. Fortuine describes *The Words of Medicine: Sources, Meanings, and Delights* (8) as a project that kept “intruding itself into my life” [p. vii] over the course of many years. Despite the title, this is not a book of medical terminology, a word list or a dictionary. He lists a number of uses for this work, but primarily he wrote it to enhance the enjoyment of the language used in the field of medicine. He tells the stories of words and puts them into context such as the imagery of medicine (heavens, earth, weather, seasons, birds, war, sports, games, toys). He goes beyond the traditional Greek and Latin foundational words and includes eponyms, abbreviations, acronyms and mnemonics. He concludes with words created in the last 50 years, such as “psychotropic”, which can be broken into its 2 parts – mind turning.

Dr. Fortuine was again named Alaska Historian of the Year in 2005 for his book, *“Must We All Die?”: Alaska’s Enduring Struggle with Tuberculosis* (9). This book is perhaps the most poignant of his works. The impact of TB in Alaska cannot be overstated. It devastated the Alaska Native population, tore children from their families and contributed to the loss of their cultures, yet its immense impact is mostly known about only within Alaska. The book’s title came from a patient interaction in the fall of 1946 when a mobile X-ray survey team visited a small Native village in interior Alaska. In this village of 70 persons, 10 active cases of TB had been identified and several other individuals had x-rays that were suspicious for active disease. Only 2 patients were treated in the hospital due to a lack of beds. When the team returned the following summer, 7 children and 2 adults had died. It was clear that others were infected, some seriously. There were no infants and no pregnant women left in the community. One villager asked a physician to see his 9-year-old son. After the child was examined, the father was told that the child had advanced pulmonary TB and would die soon. The father pleaded, “Five of my children die this way – I don’t want my other kids to go. Must we all die of the TB?” [p. xi]

In the 1920s, the ratio of TB deaths among Natives compared to whites was 11.7:1 (655 vs. 56 per 100,000). In 1931, the medical programme was transferred from the Bureau of Education in Alaska to the Office of Indian Affairs, mirroring how healthcare was delivered in the rest of the country. This brought about a 56% increase in the medical budget and saw 2 new hospitals built in rural Alaska. World War II changed Alaska due to the enormous number of military personnel stationed in the territory and the construction of roads, airfields, harbours and homes. Dr. C. Earl Albrecht, first commissioner of the Alaska Department of Health, established the Division of Tuberculosis Control in early 1946.

In 1953, a survey was conducted to collect detailed data on the disease problems facing Alaskans with a particular emphasis on Alaska Natives. The survey team was also charged with evaluating existing programmes, facilities and services. The team spent 6 weeks and interviewed 300 or so residents from all walks of life. They returned the following year and visited several remote villages. The report released in October of 1954 painted an unflinching picture of the contrast in services available to Alaska Native versus white residents. This document, known as the Parran Report, listed specific recommendations. Many of these recommendations were put into practise, strengthening the TB programmes. The recommendations were often used to justify budget increases for fighting TB and other disease conditions described in the report. A PDF copy of the report is available in the publications database found at http://www.arctichealth.org.

During the peak of the TB epidemic of the 1940s and 1950s, the Mount Edgecumbe Indian Health Service Hospital in Sitka could not afford to return deceased patients back to their families. Instead, more than 130 bodies were placed in a temporary bunker that was discovered when an expansion of the airport began in the late 1990s. Dr. Fortuine concludes his book by telling this story that began 50 years ago and only reached a conclusion in 1998.

The “story of the Sitka Mausoleum and its aftermath is a symbol of the entire story of tuberculosis in Alaska … it reminds us how a strange and deadly disease attacked the lives of many Natives and how the victims, often in the prime of life, were snatched from their homes and taken to a distant and unfamiliar place where many suffered in loneliness and died …. It tells us that Alaska is an enormous place and that transportation and communication fifty years ago were expensive, unreliable, and often simply non-existent … All the hospitals … made every effort to notify relatives of the death of a loved one … that this effort failed at times because of language barriers, distance, lost messages, or human error is regrettable, but it should not be attributed to indifference.” [p. 202]

This story has a happy ending because of the tireless efforts of a single Alaska Native helped by some 28 state and federal agencies, the airlines and many volunteers. “The present-day outpouring of leadership, cooperation, selfless effort, and good will that this incident has evoked should remind us all that these were some of the very qualities that have characterized the course of Alaska’s enduring struggle with tuberculosis.” [p. 203]

His final work, *A Century of Adventure in Northern Health: the Public Health Service Commissioned Corps in Alaska 1879–1978* (10), discusses the impact of the Public Health Service Commissioned Officers in Alaska. They have probably had a greater impact on the health of people living in Alaska compared to any other US region. This is in part due to Alaska being a federal territory from 1887 to 1959, but is also partly due to the severe and unusual health problems of Alaskans, particularly Alaska Natives. The Revenue-Cutter Service, administered by the Treasury Department was charged with intercepting contraband and protecting shipping. These ships sometimes had physicians to care for the crew and merchant seamen. In Alaska, the duties were expanded to include enforcing all US laws, conducting hydrographic surveys, protecting the fur seal herd, conducting search and rescue missions and transporting officials, criminals and the sick from one place to another. During the 1890s, they also routinely ferried reindeer from Siberia to Alaska. The book also discusses marine-hospitals, determining quarantine situations, attempting disease control, the impact of World War II and health research conducted at the Arctic Health Research Center.

## Conclusion

Robert Fortuine’s published works offer a wealth of information and insight into Alaska’s Medical History and the Health Aspects of Arctic Exploration. As is probably true for many historians, he began small, creating a bibliography and adapting a talk before tackling his first full-length book. Readers who sample his many works will be enriched and enlightened.

## Author’s note

I became aware of Dr. Fortuine’s work when he donated his files and electronically sent all of his individual bibliographies to me in 1996. As he explained in an email communication, “I have been writing on the history of medicine in the arctic regions since my medical school days. In fact, the earliest entries in my notes date from the late 1950s, when I often spent my Saturdays at the old Arctic Institute of North America Library then housed in Montreal.”

I became interested in assembling Dr. Fortuine’s wealth of bibliographic information into a single master bibliography and so spent a one-semester sabbatical in the fall of 2010 reading his major works and merging the 55 separate bibliographies into a master list. The master bibliography has over 3,000 references and 81 subjects (see the wordle image on the last page that gives a weighted visual representation of these subjects). This list has a huge number of entries for tuberculosis (almost one-third of the entries include this heading), others dwell on the history of “pre-contact” health, the history of Alaska Native health care, the history of the Alaska Department of Health, especially the tuberculosis program, the role of the U.S. Public Health Service, and traditional medicine. He completely reviewed every Governors’ and the US Surgeon General’s Reports in regard to Alaska content. This bibliography is available from the publications page found at http://www.arctichealth.org.
